# Defining conservation units in a stocking-induced genetic melting pot: unraveling native and multiple exotic genetic imprints of recent and historical secondary contact in Adriatic grayling

**DOI:** 10.1002/ece3.931

**Published:** 2014-03-18

**Authors:** Andreas Meraner, Luca Cornetti, Andrea Gandolfi

**Affiliations:** Department of Biodiversity and Molecular Ecology, Research and Innovation Centre, Fondazione Edmund MachVia E. Mach 1, 38010, San Michele all'Adige, TN, Italy

**Keywords:** European grayling, Adriatic, genetic introgression, freshwater fish conservation, Approximate Bayesian Computation

## Abstract

The definition of conservation units is crucial for the sustainable management of endangered species, though particularly challenging when recent and past anthropogenic and natural gene flow might have played a role. The conservation of the European grayling, *Thymallus thymallus*, is particularly complex in its southern distribution area, where the Adriatic evolutionary lineage is endangered by a long history of anthropogenic disturbance, intensive stocking and potentially widespread genetic introgression. We provide mtDNA sequence and microsatellite data of 683 grayling from 30 sites of Adriatic as well as Danubian and Atlantic origin. We apply Bayesian clustering and Approximate Bayesian Computation (ABC) to detect microgeographic population structure and to infer the demographic history of the Adriatic populations, to define appropriate conservation units. Varying frequencies of indigenous genetic signatures of the Adriatic grayling were revealed, spanning from marginal genetic introgression to the collapse of native gene pools. Genetic introgression involved multiple exotic source populations of Danubian and Atlantic origin, thus evidencing the negative impact of few decades of stocking. Within the Adige River system, a contact zone of western Adriatic and eastern Danubian populations was detected, with ABC analyses suggesting a historical anthropogenic origin of eastern Adige populations, most likely founded by medieval translocations. Substantial river-specific population substructure within the Adriatic grayling Evolutionary Significant Unit points to the definition of different conservation units. We finally propose a catalog of management measures, including the legal prohibition of stocking exotic grayling and the use of molecular markers in supportive- and captive-breeding programs.

## Introduction

Freshwater fish are listed among the most threatened organisms at the European level (Freyhof and Brooks 2011). Beside habitat destruction, water pollution and overharvest, the deliberate or accidental release of non-native fish is a major driver for the decline or loss of native species (Maitland 1995; Didham et al. 2007; Freyhof and Brooks 2011). Stocking and translocation of non-native taxa can have a series of detrimental effects, with competition for habitat and trophic niches, predation, and disease transfer being often reported (Vitule et al. 2009). Furthermore, the introduction of exotic taxa can lead to hybridization and varying degrees of genetic introgression, when closely related lineages are introduced and reproductive barriers are absent or incomplete (Allendorf et al. 2001).

Stocking-induced secondary contact is particularly pronounced in salmonid species. This is given by their elevated socioeconomic importance and the general practice to “enhance” declining populations with hatchery fish. In many cases, stocking practices ignored the original genetic architecture of the concerning species, thus bringing into contact isolated populations or even highly divergent phylogenetic lineages (Sušnik et al. 2004). Theoretically, stocking might provoke different genetic consequences: no admixture, when life-history traits impede hybridization (Gandolfi et al. 2006), partial admixture, when strong but incomplete isolation mechanisms are present (Meraner et al. 2010), or even the genetic breakdown of native gene pools, when no or weak isolation mechanisms are counteracting genetic introgression (Meraner et al. 2013a). However, the final genetic consequences of human-mediated secondary contact are highly unpredictable and might depend, beside on the strength of isolation mechanisms, on the immigration rate (*sensu* Hansen 2002) and stocking persistence (Marie et al. 2010).

Stocking-induced secondary contact is particularly devastating in the Mediterranean region and the northern Adriatic drainage system therein. Numerous endemic salmonid taxa are known from this zone, almost all of them suffering from genetic introgression due to stocking (Meraner et al. 2010, 2013b; but see Gandolfi et al. 2006). As an example, marble trout (*Salmo marmoratus*) suffers from widespread introgression, with few purebred populations persisting (Fumagalli et al. 2002). Even worse, Adriatic brown trout has widely been supplemented and supplanted by Atlantic Brown trout strains (Meraner et al. 2013b).

The conservation status of the Adriatic grayling (*sensu* Sušnik et al. 2004), a highly divergent genetic lineage of the European grayling (*Thymallus thymallus*), mirrors the negative population trend of this species at the European level (Uiblein et al. 2001). Most populations have been suffering from dramatic demographic declines since the last 4–5 decades due to habitat destruction, water pollution, overharvest, and bird depredation (Sušnik et al. 2004). For this reason, multiple transalpine strains of European grayling have been extensively introduced into Adriatic watercourses for stocking purposes, thus creating the conditions for population admixture. For instance, Sušnik et al. (2004) found massive and area-wide transalpine genetic introgression of Danubian (Sava River) grayling in Adriatic *T. thymallus* populations of the Soca River basin. Similarly, a detailed phylogeographic analysis of mtDNA of several *T. thymallus* populations from the northern Adriatic region revealed varying frequencies of native Adriatic haplotypes and the presence of foreign Danubian and/or Atlantic genetic variants (Meraner and Gandolfi 2012). However, native genetic variants still dominated exotic haplotypes in some stretches of the Po basin and especially in the Adige River. While these observations suggested limited genetic admixture, the study did not provide conclusive results on the occurrence and extent of hybridization (Meraner and Gandolfi 2012).

Within the Adige River basin, Meraner and Gandolfi (2012) observed intriguing mtDNA haplotype distribution patterns: while populations from the main-stem river and western tributaries were dominated by Adriatic genetic variants, samples from eastern tributaries showed no Adriatic haplotypes, but instead noticeable high frequencies of haplotypes typically occurring within the transalpine Drava River. Different explanatory scenarios were provided, spanning from recent to historical stocking and even to natural colonization through putative ancient, transalpine Adige-Drava River capture (Meraner and Gandolfi 2012). However, the limitations associated with the use of mtDNA prevented a detailed analysis of the colonization history of the eastern Adige and thus of the conservation status of its grayling populations.

Here, we provide and analyze an extensive microsatellite dataset of northern Adriatic grayling populations, with special emphasis on the Adige River basin. We include an array of *T. thymallus* reference samples from Danubian and Atlantic basins and combine nuclear genotypic data with previous as well as newly generated mtDNA sequence data. We apply model-based clustering and ABC with the following major aims:

- to define the conservation status of northern Adriatic grayling and to depict patterns and extent of stocking-induced genetic introgression;
- to trace the phylogeographic origin of non-native genetic profiles found in Adriatic populations;
- to unravel the most likely scenario of colonization history in the contact zone between eastern- and western Adige grayling populations. To this aim, we use a hierarchical ABC framework. First, we compare alternative scenarios of colonization and gene flow and, second, we estimate the approximate divergence time of eastern Adige populations;
- to propose appropriate conservation units, defined in light of the obtained data, providing a catalog of management measures aimed to protect remnant grayling populations of the northern Adriatic.

## Material and Methods

### Studied species and stocking history

**Figure 1:**
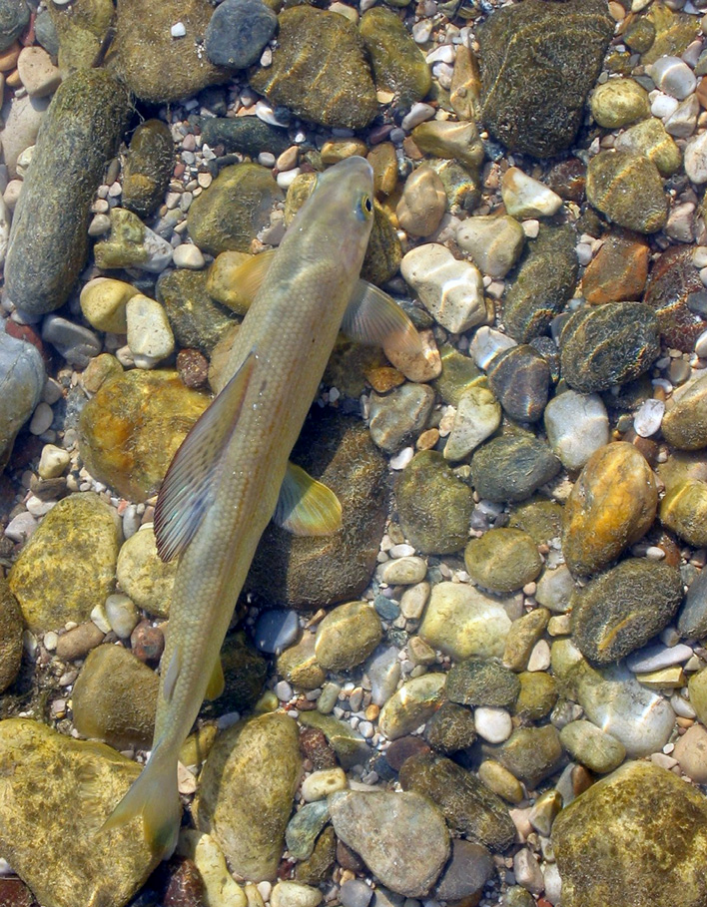
European grayling, *Thymallus thymallus*, from Adige River (Autonomous Province of Trento, Northern Italy).

European grayling (Fig. 1), *T. thymallus*, represents an originally widespread fish species of Europe, reported from the Ural Mountains to England and from Scandinavia southward to the northern Adriatic area (Northcote 1995; Kottelat and Freyhof 2007). Within its southern distribution area, *T. thymallus* populations occurred in Alpine water courses of the palaeo-Po River from Piedmont to Slovenia (Gandolfi et al. 1991; Kottelat and Freyhof 2007). However, the actual distribution of *T. thymallus* in the northern Adriatic is highly fragmentary, given the elevated sensitivity of the species to environmental degradation (Zerunian 2002). As a consequence, stocking measures have been implemented within the northern Adriatic region since last decades, using hatchery fish of transalpine origin (Bertok and Budihna 1999; Zerunian 2002; Sušnik et al. 2004). For the Adige River basin, stocking protocols documented the introduction of juvenile grayling of German, Slovenian and, recently, of Scandinavian origin (Meraner and Gandolfi 2012). Stocking density varied between river stretches and yearly inputs spanned from 1.08 kg·ha^−1^ to 5.88 kg·ha^−1^ (stocking archive 1977–2009; Fisheries Department of the Autonomous Province of Bolzano). Starting from 2012, the Autonomous Province of Bolzano (Upper Adige River Basin) firstly implemented the management implications formulated in Meraner and Gandolfi (2012) and legally prohibited stocking of grayling of transalpine origin.

### Study area and sampling strategy

**Figure 2:**
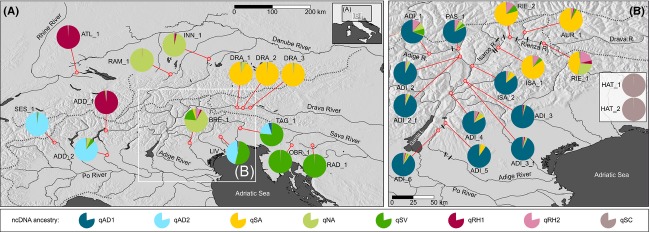
(A) Spatial distribution of test (Adriatic Drainage, except Adige Basin) and reference samples (Danube and Rhine basins) and (B) details of sampling sites within the Adige Basin. The two reference hatchery samples were collected from Italian fish farms within the Adige Basin. STRUCTURE-based mean admixture proportions at each sampling site are presented as pie charts. Impassable barriers to migration are shown in (B) as small black dash. Detailed information of sampling sites is given in Table 1.

The northern Adriatic drainage basin extends to over 100,000 km^2^ and is confined by the mountain slopes of the Alps in the north and the Apennines in the southwest. The two predominant rivers are the Po, which is the largest Italian river with a length of around 680 km and draining a watershed of 74,000 km^2^, and the Adige River, with a length of 415 km and a watershed of 12,200 km^2^. Grayling were collected from numerous northern Adriatic watercourses spanning from the Sesia River (Po Basin) in the west to Livenza and Tagliamento Rivers in the east. The core of this study was the Adige River Basin, situated in the centre of the northern Adriatic Basin (see Fig. 2).

The sample-set (*N* = 683) used in this study was obtained by merging tissue collections used in Meraner and Gandolfi (2012) and Meraner et al. (2013c). In addition, we included tissue samples from grayling larvae collected from the Adige River (*N* = 112, Table 1). This was carried out to investigate the genetic representativeness of young-of-the-year fish collections compared with adult cohorts, because the formation of captive breeding strains of *T. thymallus* will most likely be based on fry specimens.

**Table 1:** *Thymallus thymallus* samples used for genetic analyses, including sampling information.

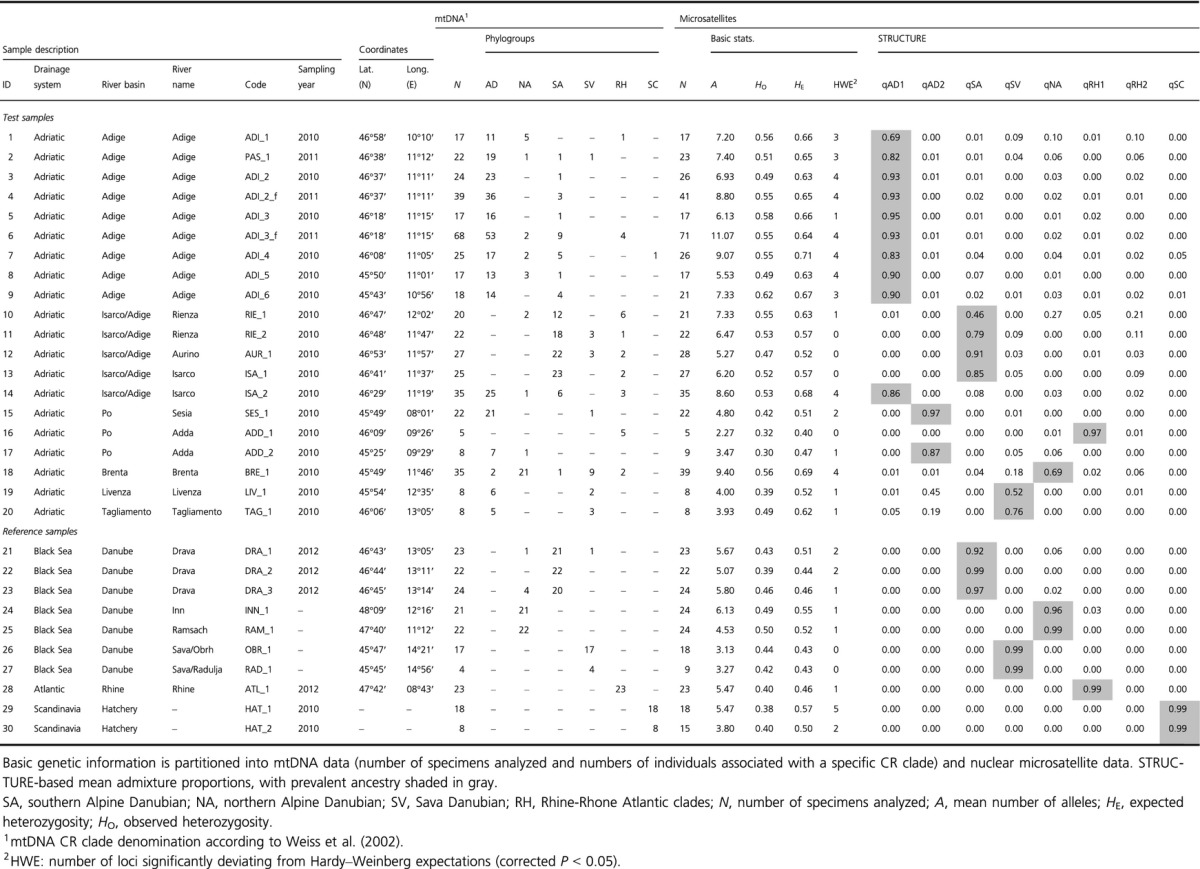

All specimens were sampled from 2010 to 2012 by riverbank or boat electro-fishing surveys, fly-fishing, or were provided by colleagues in the case of reference samples.

The sample set consisted of 20 test samples from the northern Adriatic region (*N* = 483, Table 1) and ten reference samples of northern-, southern-, Sava-Danubian, Rhine-, and hatchery origin. These reference samples (*N* = 200; Table 1) were included in order to trace the origin of non-native genetic profiles within northern Adriatic grayling populations.

### Molecular techniques

Total nucleic acid was extracted from grayling tissue samples preserved in absolute ethanol by using a standard salting out protocol (Bruford et al. 1998). Molecular screening consisted of (i) mitochondrial control region (CR) sequencing and (ii) microsatellite (SSR) genotyping.

1. The CR sequence dataset-set (*N* = 644) was achieved by merging haplotype information reported in Meraner and Gandolfi (2012) (sample IDs: 1, 3, 5, 7–20, 24, 25, 29, 30; Table 1) and Meraner et al. (2013c) (sample IDs: 21–23, 26, 27, 28; Table 1). In addition, we obtained CR sequences of 139 specimens (sample IDs: 2, 4, 6; Table 1) by adopting the methodology described in Meraner and Gandolfi (2012).
2. The microsatellite dataset (*N* = 683) consisted of a set of reference genotypes reported in Meraner et al. (2013c) (IDs: 21–28; Table 1) and newly genotyped grayling specimens (all remaining IDs; Table 1). We assayed 15 microsatellite markers: six tetranucleotide markers, namely TAR100, TAR103, TAR104, TAR108, and TAR110 (Diggs and Ardren 2008) as well as BFRO013 (Koskinen and Primmer 2001) and nine dinucleotide markers, namely BFRO004 (Snoj et al. 1999), BFRO005, BFRO007 and BFRO009 (Sušnik et al. 1999a), BFRO010 and BFRO011 (Sušnik et al. 2000), BFRO015 (Sušnik et al. 1999b), OGO2 (Olsen et al. 1998) and ONE2 (Scribner et al. 1996). Polymerase chain reaction (PCR) amplification settings and thermal profiles were the same described in Meraner et al. (2013c). Amplification products were run on an ABI Prism 3700 Genetic Analyzer (Applied Biosystems, Foster City, CA) and PCR fragments were sized and assigned to particular allele classes by using the GeneMapper® software version 4.0 (Applied Biosystems).

### mtDNA data analysis

**Figure 3:**
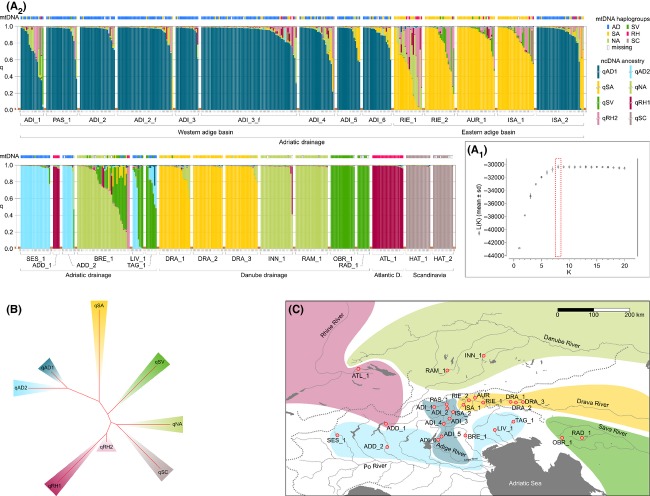
(A_1_) Mean of the estimated ln probability of data (±SD) for the different tested number of STRUCTURE genetic clusters (*K*). (A_2_) Population structure of test (first block) and reference samples (second block), ordered by drainage basins and respective subdrainages. Each vertical bar represents a fish and its proportional membership to one of the eight genetic clusters identified by STRUCTURE. In addition, mtDNA haplogroup membership is presented for each individual. Sample codes according to Table 1. (B) Neighbor-Joining tree representing the distance among clusters, based on the net nucleotide distances output by STRUCTURE. (C) A schematic representation of the geographic distribution of the genetic clusters identified by STRUCTURE (qRH2 is missing because it was under-represented in the test-samples and completely absent in the reference-samples).

All CR sequences were aligned to genetic variants presented in Meraner and Gandolfi (2012) and Meraner et al. (2013c). Among the newly sequenced specimens, we did not detect any undescribed genetic variant. CR haplotypes were grouped to major phylogenetic lineages of *T. thymallus* (*sensu* Weiss et al. 2002) building a phylogenetic tree starting from the entire CR dataset alignment (see Fig. S1). For each individual, mtDNA-lineage information is visually presented in Fig. 3, together with SSR model-based clustering results.

### Microsatellite data analysis

#### Basic statistics

Basic statistics were calculated in ARLEQUIN v3.5.1.2 (Excoffier and Lischer 2010) and included number of alleles (*A*), expected (*H*_E_) and observed (*H*_O_) heterozygosities, departure from Hardy–Weinberg (HW) expectations (subsequently tested by a Fisher's exact test; Guo and Thompson 1992), and linkage equilibrium (Chi-square test; Slatkin and Excoffier 1996). The presence of null alleles was tested with MICRO-CHECKER (Van Oosterhout et al. 2004). Significance of all multiple statistical tests was corrected by using the false discovery rate (FDR) method implemented in the software QVALUE (Storey 2002).

#### Model-based clustering

Population structure and genetic introgression of 683 grayling specimens from 30 sampling sites were investigated using STRUCTURE 2.3.2.1 (Pritchard et al. 2000), which uses a Bayesian approach to cluster specimens based on the minimization of genetic linkage and Hardy–Weinberg (HW) disequilibria (model parameters: tested from *K* = 1 to *K* = 20 genetic clusters, each with twenty replicates consisting of 500,000 burn-in and 1,000,000 markov chains; admixture model with independent allele frequencies).

The most likely number of genetic clusters (*K*) represented by our grayling dataset was estimated using the methodology presented in Pritchard et al. (2000) and the ad hoc statistic “estimated Ln Prob of Data,” because the value of *K* that maximizes the estimated model ln-likelihood, ln(*P*(*X*|*K*)), is a sensible choice for the number of clusters presented by the dataset (Falush et al. 2003). For the most likely value of *K*, convergence of the 20 independent replicate runs was checked. As no full convergence was observed and slightly different clustering solutions were observed, a majority-rule approach was applied to select a single and most informative clustering solution. Individual admixture proportions (*q*-values; i.e., the estimated membership coefficients for each individual in each cluster) and population-level admixture estimates (*Q*-values; i.e., the mean of individual *q*-values for a given sample) were taken from a single run among replicates converging to the selected clustering solution.

Intracluster genetic substructure was analyzed by independently re-running STRUCTURE for each of the inferred genetic clusters (model parameters as described above, but limiting from *K* = 1 to *K* = 5 and using both the “independent-” and the “correlated allele frequency” options). Thereby, only individuals with a *q*-value ≥0.90 for a defined cluster were included in these analyses.

### Inference of colonization history of *T. thymallus* within the Adige River basin

#### ABC model set-up

**Figure 4:**
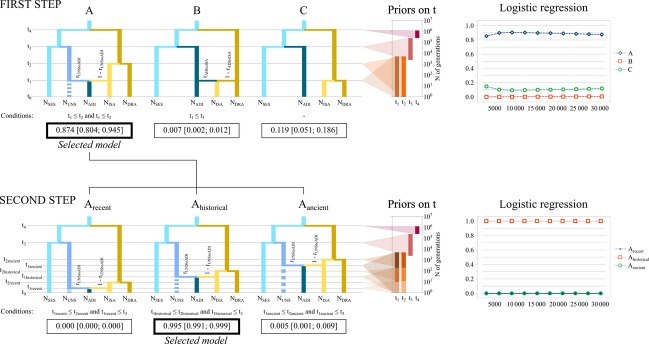
Graphic representation of the hierarchical ABC analysis, with three scenarios tested for each of the two steps. Relative posterior probabilities, obtained through a logistic regression computed every 10% of the number of 30,000 simulated data closest to the observed data, are presented for scenarios A, B, and C (top right) and for *A*_recent_, *A*_historical_, and *A*_ancient_ (bottom right). Posterior probability, computed on 30,000 simulated data closest to the observed data, and 95% confidence interval are shown for each scenario. Populations codes: SES = Sesia; UNS = unsampled population; ADI = Adige; ISA = Isarco; DRA = Drava. *r*_XtoY_ (rate of admixture) represents the contribution of a population *X* to the origin of a new admixed population *Y*, ranging from 0 to 1.

Approximate Bayesian Computation (ABC; Beaumont et al. 2002) is a family of statistical techniques to perform parameter estimation and model selection, increasingly used in ecology and evolution to make inferences about complex evolutionary scenarios, bypassing direct estimates of likelihood function and adopting comparison between observed and simulated summary statistics (Cornuet et al. 2010). To depict the most likely colonization scenario for eastern Adige and the potential genetic contribution to western Adige populations through gene flow, we applied ABC as implemented in the DIYABC v.1.0.4 package (Cornuet et al. 2008, 2010). Firstly, we defined four meta-populations: three from Adriatic and one from Danubian water courses. The data from 12 microsatellite markers following a generalized stepwise mutation model were retained (TAR100, TAR104 and TAR110 were excluded because containing imperfect, i.e., non-strictly tetranucleotidic, repeats). As signals of genetic introgression from recently stocked specimens had been detected in all sampling sites by the STRUCTURE analysis, for each cluster, we selected individuals with *q*-values >0.90 only, and defined samples SES (Po basin, Adriatic, *N* = 20) from SES_1, ADI (western Adige basin, Adriatic, *N* = 40), by pooling individuals from ADI_1, ADI_2, ADI_3, ADI_4, ADI_5, and ADI_6, ISA (eastern Adige basin, Adriatic, *N* = 39), by pooling individuals from RIE_1, RIE_2, ISA_1, and AUR_1, and DRA (Drava basin, Danubian, *N* = 40) by pooling individuals from DRA_1, DRA_2 and DRA_3 (Fig. 4).

Following the results of the model-based clustering and mtDNA analyses, we applied the following ABC approach (Fig. 4):

1. we first defined three candidate demographic models (Fig. 4): (A) ISA derives from DRA and ADI derives from admixture of ISA and an unsampled Adriatic population (UNS) (i.e., unidirectional gene flow from eastern to western Adige), (B) ISA derives from admixture of DRA and ADI (i.e., unidirectional gene flow from western to eastern Adige) and (C) ISA derives from DRA while ADI from SES (i.e., no gene flow between western and eastern Adige).
2. in a second step, we defined three submodels from the model selected in step (i), each differing from the others in the time of split of populations ISA and DRA (Fig. 4). Submodel (*A*_recent_) assumed a recent origin of ISA (1 ≤ *t*_2recent_ ≤ 10 generations), subsequent to the onset of stocking measures after 1970; submodel (*A*_historical_) assumed an intermediate time frame for ISA formation (11 ≤ *t*_2historical_ ≤ 200 generations), in accordance with the hypothesis of medieval stocking, while the third submodel assumed natural colonization following an ancient, transalpine river shortcut (*A*_ancient_) (201 ≤ *t*_2ancient_ ≤ 25,000 generations) (Fig. 4).

We simulated 1 × 10^6^ datasets for each of the six scenarios, building reference tables based on a combination of six summary statistics, namely NAL, MGW, FST, LIK, V2P, and DAS (see Table S1; for a full description see DIYABC documentation available from: http://www1.montpellier.inra.fr/CBGP/diyabc/). The priors for all demographic and mutation model parameters, namely the time of split or admixture events (*t*_n_), the effective population size (*N*_X_), the rate of admixture (*r*_XtoY_), the mean mutation rate (*μ*_SSR_), and the mean parameter of the geometric distribution of the length in number of repeats of mutation events (*P*_SSR_) had uniform distribution defined by minimum and maximum values. Prior distributions of all parameters are described in Table S2. We applied a conservative mean generation time for grayling of *g* = 4 years (the lowest estimate by Haugen and Vøllestad 2001; probably most realistic for southern European populations). One percent of the simulated datasets with summary statistics most similar to the observed data were then used to conduct a logistic regression in order to estimate the posterior probability of each scenario and its 95% credibility intervals, as proposed by Cornuet et al. (2008, 2010).

We finally calculated posterior distribution of each demographic parameter in each scenario using a logistic regression, after applying a ln-tg transformation to parameters values (see Table S3).

#### ABC model checking

We evaluated the “goodness-of-fit” of all evolutionary scenarios by using the “model checking” option in DIYABC v.1.0.4 aimed to measure the discrepancy between a combination of a model and its parameter posterior distributions to a “real” dataset (Cornuet et al. 2010). We followed Cornuet et al. (2010) and based model checking on summary statistics not applied for model selection (i.e., HET, VAR, DM2, N2P, H2P; see Table S1 and DIYABC documentation for details). Finally, we simulated 500 datasets per model with parameter values drawn from the same prior distributions as the models to calculate: (i) the proportion of cases that the selected scenario had not the highest posterior probability among the three competing ones, when it was the true scenario (type I error, estimated from test datasets simulated under selected scenario); (ii) the proportion of cases that the selected scenario had highest posterior probability when it was not the true one (type II error, estimated from cumulative test datasets simulated under alternative scenarios).

## Results

### mtDNA

A total of 41 haplotypes were resolved from the alignment of CR sequences of 644 individuals (83 haplotypes, excluding congeneric outgroups and when merging previously published undetected and new mtDNA sequence results) (Fig. S1). Overall, phylogenetic analyses confirmed the general pattern presented in Weiss et al. (2002), with seven major evolutionary lineages partitioned into Danubian (northern Alpine Danubian [NA], southern Alpine Danubian [SA], and Sava Danubian [SV]), Atlantic (Mixed Central Europe [RH], Rhine-Danubian [RD], Scandinavian [SC]), and Adriatic [AD] main groups (Fig. S1).

AD haplotypes were found at 15 of 20 northern Adriatic sampling sites, including newly sequenced fry collections from the Adige River (AD ratios between 0.06 and 0.96, Table 1), but in all cases, they were found together with Danubian and/or Atlantic haplotypes. In contrast, Upper Adda River samples (ADD_2) were fixed for RH and eastern Adige sites (RIE_1, RIE_2, AUR_1 and ISA_1) harbored predominantly SA haplotypes (Table 1). Haplotype assemblage of reference samples was mostly in line with the hydrogeographic origin of the respective samples: SA haplotypes predominated in Drava River samples, NA were fixed in Inn and Ramsach Rivers, SV in Sava River tributaries, while Rhine grayling were fixed for RH haplotypes. Finally, grayling sampled from Italian fish farms and used for re-stocking in 2011–2012 were fixed for the SC phylogenetic lineage (Table 1).

### Microsatellites – basic statistics

Overall, number of alleles per locus ranged from five, for marker BFRO007, to 53, for marker TAR110. At the population-level, the number of alleles showed a minimum value for the sample ADD_1 (*A* = 2.27) and a maximum for the sample ADI_3_f (*A* = 11.07), with an across-population mean value of 5.98 (Table 1). Expected heterozygosities (*H*_E_) ranged from 0.40 at ADD_1 to 0.71 at sampling site ADI_4. Departures from HW expectations were statistically significant after false discovery rate (FDR) correction in 60 of 450 cases (13.3%). Three loci, namely BFRO009, TAR110, and TAR104, showed cumulated significant heterozygosity excess and MICRO-CHECKER results indicated the possible presence of null alleles. Therefore, we tested the effect of these loci on model-based clustering, which is known to be sensitive to HW pattern. We run STRUCTURE either with or without these three loci, setting *K* = 8 as the most likely number of genetic clusters. Both analyses gave compatible results, and thus, we retained the original dataset based on all 15 microsatellite loci (ΔQ = difference of population-level admixture between datasets based on 15 and 12 loci, respectively; mean observed ΔQ = 0.01; 0 < ΔQ < 0.10 for each of eight genetic clusters).

No pair of loci evidenced deviations from linkage equilibrium, thus suggesting that all 15 loci are unlinked (data not shown).

### Population structure and genetic introgression

STRUCTURE-based assignment analysis detected eight distinct genetic clusters, supported by a plateau phase reached at *K* = 8 for the statistic “estimated Ln Prob of Data” (Fig. 3A_1_).

The reference samples of Danubian or Atlantic origin (Table 1; Figs. 2, 3) were mostly identified by single genetic clusters, with only marginal signatures of trans-basin genetic introgression: Drava River samples (DRA_1, DRA_2 and DRA_3, Table 1) were predominated by SA mtDNA haplotypes, with the associated nuclear genetic cluster qSA reaching *Q*-value frequencies from 0.92 to 0.99 at single localities (Table 1; Figs. 2, 3). Inn and Ramsach River samples (INN_1, RAM_1) were dominated by NA mtDNA haplotypes and the associated nuclear genetic cluster qNA (0.96 ≤ qNA ≤ 0.99) (Table 1; Figs. 2, 3). Sava River samples (OBR_1 and RAD_1) harbored SV mtDNA genetic variants, with the associated nuclear genetic cluster qSV reaching 0.99 (Table 1; Figs. 2, 3). The Upper Rhine River sample (ATL_1) was fixed for the RH mtDNA clade, thus referring the corresponding nuclear genetic cluster as to qRH1 (qRH1 = 0.99) (Table 1; Figs. 2, 3). Finally, hatchery grayling sampled from Italian fish farms (HAT_1 and HAT_2) were all fixed for SC mtDNA haplotypes, thus referring the according genetic cluster as to qSC (qSC = 0.99) (Table 1; Figs. 2, 3).

Distribution patterns of nuclear genetic clusters within northern Adriatic samples were more complex; AD mtDNA haplotypes were predominately linked with two distinct, but closely related, nuclear genetic clusters, referred to as qAD1 and qAD2 (Figs. 2, 3). Cluster qAD1 was widely distributed within the Adige drainage basin and was found at all ADI_ samples, at PAS_1, as well as at ISA_2, which is located at the confluence of Isarco and Adige Rivers. Population-level admixture proportions of qAD1 ranged from 0.69 at ADI_1 to 0.95 at ADI_3 (Table 1). A second Adriatic genetic cluster, qAD2, was found in several Adriatic samples but not in the Adige River, with *Q*-values ranging from 0.19 at TAG_1 to 0.97 at SES_1. Noteworthy, sample sets were never entirely fixed for qAD1 or qAD2 genetic clusters, and multiple signatures of trans-basin Danubian and/or Atlantic grayling gene pools, either of purebred exotic or of hybrid origin, were detected within all samples from Adriatic water courses. These exotic genetic signatures resembled those found in reference samples, specifically in qSA, qSV, qNA, qRH1, and qSC (Table 1; Figs. 2, 3). However, a sixth exotic genetic cluster not represented by our reference dataset was detected within the northern Adriatic samples. This cluster was found in three purebred specimens, one found at RIE_1 and two at BRE_1 samples, as well as in several hybrid fish of the Adige River samples. This genetic cluster was closely linked to qRH1 in STRUCTURE-based distance tree (Fig. 3B), was associated with RH mtDNA genetic variants, that is, haplotype At28 in the case of purebred specimens, and was thus referred to as qRH2. Moreover, some samples (e.g., ADI_1 and TAG_1) showed exceptionally high levels of non-AD genetic signatures, while at samples BRE_1 (predominance of qNA) and ADD_1 (fixed for qRH1), no native qAD genetic profiles were detected at all. Finally, samples collected from rivers belonging to the eastern Adige basin (RIE_1, RIE_2, AUR_1 and ISA_1) showed no qAD genetic signatures but were predominated by varying frequencies of qSA membership, spanning from 0.46 at RIE_1 to 0.91 at AUR_1 (Table 1; Figs. 2, 3).

STRUCTURE-based analysis of genetic substructure gave similar results when comparing assignment tests run under either the independent or the correlated allele frequency options (Fig. S2): no genetic substructure was revealed within clusters qAD1, qSC, and qSV. In contrast, substantial genetic substructure was found within clusters qAD2, qRH1 as well as qSA. In detail, within qAD2, fish from Sesia (SES_1) were separated from Lower Adda (ADD_2) as well as from Livenza (LIV_1) samples, while within qRH1, Upper Adda samples (ADD_1) were discriminated from Rhine (ATL_1) River samples. Finally, Drava River fish (DRA) were clearly separated from eastern Adige grayling populations (RIE_1, RIE_2, AUR_1 and ISA_1) (Fig. S2).

### Colonization history of *T. thymallus* within the Adige River

The evolutionary history of eastern Adige populations and their possible contribution to the distinct western Adige populations through migration and gene flow was investigated by a hierarchical ABC analysis: on a first level of analysis, the most likely evolutionary scenario suggested that the eastern Adige population (ISA) derives from the Drava (DRA), while the western Adige population (ADI) originates from admixture of ISA and a hypothetical ancestral Adriatic grayling population (model [A] with posterior probability = 0.8747 [0.8042 ≤ CI ≤ 0.9453]). We thus selected model (A) and inferred the divergence time of population ISA from DRA as well as the admixture rate of ISA into ADI. Submodel *A*_historical_, with an intermediate prior on time since divergence of ISA from DRA (11 ≤ *t*_2historical_ ≤ 200), was highlighted as the most likely scenario (posterior probability of submodel *A*_historical_ = 0.9995 [0.9991 ≤ CI ≤ 0.9999]), with a median divergence time between ISA and DRA estimated at *t*_2historical_ = 116 generations and a median admixture rate of the ISA gene-pool into ADI (i.e., gene flow from eastern to western Adige; 1 – *r*_UNStoADI_ in Table S3) estimated at 0.174.

Model checking of both first- and second-level ABC analyses and when applying summary statistics not used for model discrimination supported the selected models as the “best” evolutionary scenarios. In the first-level analysis, the type I error rate was 0.29, and the type II error rate was 0.11, while in the second-level analysis, the type I error rate was 0.08, and the type II error rate was 0.02.

Virtually, the same results were obtained repeating the analysis after the exclusion of marker BFRO009, showing significant deviation from HWE (data not shown).

## Discussion

### Conservation status of Adriatic grayling

While numerous genetic studies investigated northern and central European populations of *T. thymallus* (see Gum et al. 2009), molecular data concerning the conservation status and population structure of Adriatic populations have been scarce so far. Sušnik et al. (2004) genotyped grayling of the Soca River basin in Slovenia and detected massive genetic introgression of Danubian origin in all populations, with only few potentially purebred Adriatic grayling specimens persisting. The authors concluded that, given the alarming conservation status and the evolutionary distinctness of the Adriatic grayling, any non-introgressed (sub)population should have a high priority for conservation. Likewise, the official “Red List of Threatened Species” (IUCN) acknowledged the particular threat status of Adriatic *T. thymallus* but concluded that “because of extensive stocking throughout the northern Adriatic basin it might be too late to investigate [its] original morphology, variability, genetics, and distribution” (Freyhof 2011). In contrast to the staggering Slovenian situation, Meraner and Gandolfi (2012) highlighted unexpectedly high frequencies of mtDNA haplotypes of the indigenous Adriatic phylogroup, exceeding 90% in several river stretches of the Po and the Adige basins. While indicative of a persistence of native gene pools; however, only a thorough analysis of nuclear DNA could have unraveled the degree of genetic introgression and hybridization in these populations.

In the present study, two genetically distinct Adriatic clusters were identified in 14 of 20 Adriatic sampling sites, named qAD1 and qAD2. Cluster qAD1 was widely distributed among sampling sites within the western Adige basin and exceeded population-level admixture proportions of 0.90 in several sampling localities. This is an important and encouraging outcome in terms of conservation and is in sharp contrast to the widespread introgression scenario reported by Sušnik et al. (2004), where only 50–60% of the original Adriatic gene pool persisted. Within the Adige genetic cluster, qAD1, no substructure was identified, suggesting a uniform grayling metapopulation within the western Adige basin.

Cluster qAD2 was encountered within the Adriatic basin at four sampling sites (SES_1, ADD_2, LIV_1, and TAG_1) outside the Adige River, with frequencies ranging from 0.19 at Tagliamento River (TAG_1) to a maximum of 0.97 at Sesia River (SES_1) (Table 1; Figs. 2, 3). In contrast to qAD1, substantial genetic substructure was detected within qAD2, separating Sesia (SES_1) samples from those of Lower Adda (ADD_2) as well as from those of the Livenza River (LIV_1). These findings suggest pronounced microgeographic population structure of Adriatic grayling populations inhabiting distinct northern Adriatic rivers. Po River tributaries have been separated from eastern Adriatic rivers because of the re-rise of the Adriatic sea-level around the end of the last glacial period (see Salzburger et al. 2003), thus isolating their grayling populations and favoring population divergence for at least approximately 2000 generations. On the other hand, genetic divergence of SES_1 versus ADD_2, which are hydrologically connected through the Po River main course, could reflect strong homing behavior, low dispersal ability (see Swatdipong et al. 2010), and/or ecological isolation. In fact, *T. thymallus* is a strictly cold-water and high-oxygen demanding species (Northcote 1995). These features could impede down-stream migration into the Po main course and could thus prevent gene flow between adjacent tributaries of the Po. Finally, even anthropogenic barriers to migration (e.g., weirs and dams), fragmenting middle and lower courses of both Adda and Sesia Rivers, might play a role in promoting genetic divergence between SES_1 and ADD_2 samples, as previously demonstrated for Central European *T. thymallus* populations (Meldgaard et al. 2003).

### Patterns and extent of stocking-induced genetic introgression

Beside indigenous Adriatic genetic signatures, multiple exotic genetic patterns were encountered in northern Adriatic grayling populations (see Table 1; Figs. 2, 3). Genetic imprints of Danubian- and Atlantic genetic clusters were scattered at varying degrees among all Adriatic sampling sites and likely reflect a shifting genetic composition of grayling hatchery stocks during the last few decades. In contrast to brown trout, *Salmo trutta*, for which permanent Atlantic captive breeding strains have been established over dozens of generations and stocked across Europe (Meraner et al. 2013b), for grayling transalpine hatchery stocks mostly derive from wild spawners (Baars et al. 2001; Meraner et al. 2013c; G. Unfer personal communication). This breeding scheme increases the risk of introducing multiple highly divergent grayling stocks, especially when spawners are transferred from north to south from different geographically remote donor populations. Such a temporally unstable genetic composition of stocked grayling is supported by available stocking protocols (Meraner and Gandolfi 2012; fisheries archive of the Autonomous Province of Bolzano) and by the present genetic data, which revealed multiple genetic signatures of Danubian and Atlantic origin. These exotic genetic patterns are still rather marginal in several Adriatic sampling sites where populations are still dominated by native Adriatic genetic clusters (qAD1 or qAD2), and only a low number of fish of completely exogenous or hybrid origin is present. However, at single sites, we detected massive hybridization from multiple exogenous sources. As an example, the native qAD1 cluster accounts only for 0.69 in the Upper Adige (ADI_1), where introgression is widespread from Danubian (qNA, qSV) and Atlantic (qRH2) source populations. The situation is even worse at site BRE_1 (Brenta; Table 1), where traces of an original Adriatic grayling population were detected at the mtDNA level but were absent at the nuclear level (Table 1; Figs. 2, 3). At BRE_1, the current grayling population is composed by individuals with genetic composition largely related to the Danubian qNA cluster, with additional genetic contributions from qSV, qRH1, and qRH2. Finally, a complete genomic replacement of a hypothetical former Adriatic grayling population by the Atlantic genetic cluster qRH1 was detected within the Adda River at site ADD_1, situated close to the Italian/Swiss border. This “Atlantic” grayling population may have been originated from stocking of grayling from dammed Lake Livigno, which itself is thought to have received grayling from Upper Inn River (V. Adami, pers. comm.). Although the Inn River is part of the Danubian basin, it has recently been shown to hold grayling of Atlantic origin (Weiss et al. 2013).

The different genetic effect of stocking at single sampling sites, spanning from almost no introgression to the complete collapse of the native population, might be linked to different “immigration rates” of stocked fish (*sensu* Hansen 2002). Specifically, for grayling, different amounts of genetic introgression might reflect microgeographic discrepancies of annual input of introduced fish and/or stocking duration (Meraner and Gandolfi 2012). Such a scenario is supported for the Adige River situation, where the most heavily introgressed population (ADI_1) is also the one which received massive stocking load (maximum annual biomass input = 5.49 kg·ha^−1^, 27 out of the last 33 years with stocking), while downstream sites along the Adige River (e.g., ADI_2) were only moderately stocked (biomass input = 1.58 kg·ha^−1^; seven out of 33 years with stocking).

### Evolutionary history of *T. thymallus* of the eastern Adige River basin

Puzzlingly and in contrast to its hydrogeographic location, eastern Adige grayling was predominated by the cluster qSA, typically found in Danubian Drava River populations, while Adriatic genetic signatures (either on the mtDNA or the ncDNA level) were completely missing. Both STRUCTURE analysis and ABC rejected the hypothesis of a genetic contribution of the Adriatic genetic cluster (qAD1) in eastern Adige. The two analyses instead supported a Danubian origin of eastern Adige populations, with likely limited unidirectional gene flow occurring from eastern to western Adige populations. So forth, eastern Adige populations are, however, unlikely to stem from recent stocking measures. ABC rejected a recent colonization scenario and suggested a divergence time of eastern Adige from Drava populations having occurred several hundreds of years ago. Both posterior probabilities of model selection (*P* = 0.8747 for model A in step 1, *P* = 0.9995 for submodel *A*_historical_ in step 2), as well as model checking, gave high statistical support for such colonization scenarios, and indicate a historical transalpine origin of grayling inhabiting eastern Adige River stretches (see Fig. 4). Intriguingly, ABC estimated a median divergence time of 116 generations ago, which would place the origin of eastern Adige populations very close to the ruling period of the Austrian monarch Maximilian I (1486–1519). Maximilian I is known to have conducted first salmonid stocking in Austrian waters originally free of fish (Pechlaner 1984; Weiss et al. 2001), and he is also supposed to have encouraged transalpine fish transport from Austrian to northern Italian waters (Meraner et al. 2007; Meraner and Gandolfi 2012).

Clearly, the estimation of divergence time through ABC has to be interpreted with caution, because the determination of parameter priors might critically affect analysis accuracy. However, close concurrence of the formerly proposed hypothesis of historical stocking with independent Bayesian calculations makes medieval grayling transfer indeed the most likely scenario, explaining the actual predominance of Danubian grayling in the eastern Adige basin. In contrast to present-day usage, historical stocking is thought to have been applied exclusively to establish new fisheries in formerly fish-free alpine areas (e.g., genera *Salmo* and *Salvelinus* in high-alpine lakes; Pechlaner 1984) and not to augment fisheries yield of wild populations. Thus, the introduction of Danubian strains into eastern Adige basin seems reasonable only under a scenario of grayling being absent from the eastern Adige basin prior to historical stocking. Two scenarios seem plausible: (i) grayling did never naturally colonize the eastern Adige basin; rather, steep and cliffy stretches of the middle Isarco River (zone between ISA_1 and ISA_2 in Fig. 2B) could have impeded grayling immigration from a hypothetical glacial refuge in the northern Adriatic zone into the eastern Adige basin. (ii) Alternatively, grayling could have colonized eastern Adige basin from Adriatic source populations but underwent extinction in medieval times in these eastern zones, thus motivating subsequent stocking. Historical mining activities in the eastern Adige basin, such as intensive copper-mining in the Aurino River valley (e.g., upstream to sampling site AUR_1 in Fig. 2B; Kolb and Mayer 2010) could potentially have induced heavy-metal pollution of watercourses belonging to the eastern Adige basin and could have eradicated grayling populations therein. Indeed, this scenario of historical mining-induced fish extirpation remains speculative for the area of interest, due to the lack of concrete and provable evidences. However, there is growing scientific evidence that historical mining-induced heavy-metal load represents a largely underestimated problem in the context of environmental pollution (Bindler et al. 2009).

### Management implications and conclusions

The results underline the persistence of unexpected high frequencies of indigenous genetic profiles in several Adriatic watercourses. However, our data also highlight recent genetic introgression from multiple exotic source populations and suggest, for the eastern Adige, historical stocking.

In order to prevent further loss of indigenous Adriatic grayling populations, it is thus clear that a strong action should be taken to prevent the negative effects of stocking on native Adriatic grayling biodiversity. Such fisheries practices have been banned for the Upper Adige since 2012 but are still ongoing in most other regions of the Adriatic area. Thus, an immediate and strict areawide prohibition of stocking exotic grayling is crucial in terms of conservation. To this aim, genetic evidences of the malign impact of stocking have to be promptly transmitted to fisheries authorities. Thereby efficient ways of communication have to be incited, including round table discussions and popular scientific articles published in fisheries and conservation magazines, where scientific jargon is avoided and clear key conservation messages are highlighted (for communication in fisheries science see also: Dedual et al. 2013).

The abandon of introducing transalpine grayling will demand the formation of supportive breeding or captive breeding strains of native Adriatic grayling. This is particularly relevant for those populations found in river stretches where natural reproduction is hindered by hydraulic engineering and hydroelectric use. Given area-wide genetic introgression, genetic monitoring and marker-assisted selection of suitable indigenous spawners will be inevitable to impede further progression of genetic introgression and reduce the risk of “forced matings” of native and exotic fish in the hatchery environment. Most likely, captive breeding of Adriatic grayling will depend on fry specimens, as adult grayling can hardly be acclimated to the hatchery environment (G. Unfer, pers. comm.). Genetic monitoring of young-of-the-year grayling (ADI_2f and ADI_3f; Table 1) evidenced limited genetic introgression in these cohorts, thus representing a suitable source for the establishment of indigenous breeding strains suitable for future restocking initiatives.

It is important to underline that there is not a single uniform gene pool of the Adriatic Grayling, but a pronounced microgeographic genetic substructure was demonstrated within this “Evolutionary Significant Unit” (ESU; *sensu* Moritz 1994). We found significant genetic substructure among Adriatic rivers, suggesting that probably all major Adriatic watercourses hold unique gene pools, definable as distinct “Management Units” (MU; *sensu* Moritz 1994) potentially associated with ecological differences and adaptive value (Koskinen et al. 2002), thus deserving particular protection.

The situation of MUs is particularly complex within the Adige River basin, where a historical contact zone with substantial geneflow from east to west has to be assumed. Therein, two distinct management units should be defined and conserved: an “Adriatic” gene pool in western and main-stem Adige and a “Danubian” metapopulation in the eastern part of the Adige River. Grayling populations of eastern Adige are most likely of anthropogenic origin and thus do not meet the international criteria of “native” gene pools (Manchester and Bullock 2000). However, genetic data suggest that these eastern Adige grayling populations have been formed several hundreds of years ago and would thus meet the Italian legislative criteria of “para-autochthonous” gene pools (see AA VV 2007). Beside legislative considerations, the conservation of “Danubian” grayling in the eastern Adige basin seems thoughtful, given the complete absence of an Adriatic genetic counterpart in these rivers as well as the self-sustainability of these stocks, important also for local fisheries.

## Supporting Information

Details on model checking for the alternative scenarios of colonization and gene flow tested in the two steps ABC analysis (Table S1), prior distribution of temporal, demographic and mutation model parameters used in the two steps of ABC analysis (Table S1), parameter posterior distributions inferred by ABC under the *A* and *A*_historical_ scenarios, selected in first and second step, respectively (Table S3), a midpoint-rooted Maximum Likelihood Phylogeny of 83 *T. thymallus* mtDNA CR haplotypes based on the ML algorithm (Fig. S1) and intracluster genetic substructure analysis results (Fig. S2) are provided, together with supplementary references, as Supporting information. Microsatellite dataset, with individual information about sampling location and GenBank reference for mtDNA control region, mtDNA sequence alignment used to generate the phylogenetic tree presented in Fig. S1 and microsatellite dataset analyzed in ABC are available from the Dryad Digital Repository: http://doi.org/10.5061/ dryad.p2f7c.

**Table S1**. Model checking for the alternative scenarios of colonization and gene flow tested in the two steps ABC analysis. **Table S2**. Prior distribution of temporal, demographic, and mutation model parameters used in the two steps of ABC analysis. **Table S3**. Parameter posterior distributions inferred by ABC under the *A* and *A*_historical_ scenarios, respectively, selected in first and second step. **Figure S1**. Midpoint-rooted Maximum Likelihood Phylogeny of 83 *T. thymallus* mtDNA CR haplotypes based on the ML algorithm. **Figure S2**. Intracluster genetic substructure analysis results.
