# Supplementary material for: Defining conservation units in a stocking-induced genetic melting pot: unraveling native and multiple exotic genetic imprints of recent and historical secondary contact in Adriatic grayling
Source: Ecol Evol. 2014 Mar 18;4(8):1313–27. doi: 10.1002/ece3.931 (PMC4020691; doi:10.1002/ece3.931)
Supplement: Supplementary file 1 — Table S1. Model checking for the alternative scenarios of colonization and gene flow tested in the two steps ABC analysis. Table S2. Prior distribution of temporal, demographic, and mutation model parameters used in the two steps of ABC analysis. Table S3. Parameter posterior distributions inferred by ABC under the A and Ahistorical scenarios, respectively, selected in first and second step. Figure S1. Midpoint-rooted Maximum Likelihood Phylogeny of 83 T. thymallus mtDNA CR haplotypes based on the ML algorithm. Figure S2. Intracluster genetic substructure analysis results. [file ece30004-1313-sd1.pdf]

Supporting information

Table S1 Model checking for the alternative scenarios of colonisation and gene-flow tested in the two steps ABC analysis. For each Summary statistics (S), the probability Prob ($S_{simulated} < S_{observed}$) was computed from 10,000 data sets simulated from the posterior distributions of parameters obtained under a given scenario. Corresponding tail-area probabilities (p-values) of each summary statistic can be obtained as Prob ($S_{simulated} < S_{observed}$) and 1.0 - Prob ($S_{simulated} < S_{observed}$) for Prob ($S_{simulated} < S_{observed}$) ≤ 0.5 and > 0.5, respectively (Gelman *et al.* 1995). Summary statistics, in both first and second step, are divided into 'used' to discriminate among scenarios and compute parameter posterior distributions (a), and 'not used' for model selection but used in model checking (b). Significant tail-area probabilities, after applying the false discovery rate correction method (FDR) implemented in the software QVALUE (Storey 2002), are marked with asterisks (*, **, *** = tail-area probability < 0.05, < 0.01 and < 0.001, respectively). Populations codes: SES = Sesia; DRA = Drava; ISA = Isarco; ADI = Adige. Summary statistics codes: NAL_i = mean number of alleles in population i; MGW_i = mean ratio of the number of alleles over the range of allele sizes (Garza & Williamson 2001); FST_i-j = F_{ST} value between populations i and j (Weir & Cockerham 1984); LIK_i-j = mean individual assignment likelihoods of population i assigned to population j (Pascual *et al.* 2007); V2P_i-j = mean variance of the absolute allelic size pooling samples from populations i and j; DAS_i-j = shared allele distance between populations i and j (Jin & Chakraborty 1994); HET_i = mean expected heterozygosity in population i (Nei 1987); VAR_i = mean allelic size variance in population i; DM2_i-j = delta mu squared (Goldstein *et al.* 1995); N2P_i-j = mean number of alleles pooling samples from populations i and j; H2P_i-j = mean expected heterozygosity pooling samples from populations i and j.

Table S1

(a)	Summary statistics	observ ed value	A	B	C	A_{recent}	$A_{historical}$	$A_{ancient}$
Posterior probability [C.I.]			0.874 [0.804; 0.945]	0.007 [0.002; 0.012]	0.119 [0.051; 0.186]	0.000 [0.000; 0.000]	0.995 [0.991; 0.999]	0.005 [0.001; 0.009]
Summary statistics used to discriminate among scenarios and compute parameter posterior distributions	NAL_SES	3.750	0.738	0.740	0.739	0.739	0.739	0.739
	NAL_DRA	3.500	0.600	0.600	0.600	0.599	0.599	0.599
	NAL_ISA	3.917	0.693	0.685	0.695	0.718	0.746	0.696
	NAL_ADI	5.000	0.861	0.875	0.875	0.506	0.641	0.820
	MGW_SES	0.410	0.008 (*)	0.008 (*)	0.008 (*)	0.008 (*)	0.008 (*)	0.008 (*)
	MGW_DRA	0.527	0.055	0.056	0.056	0.056	0.055	0.056
	MGW_ISA	0.568	0.094	0.154	0.093	0.099	0.103	0.092
	MGW_ADI	0.436	0.090	0.011 (*)	0.011 (*)	0.909	0.891	0.328
	FST_SES-DRA	0.607	0.419	0.419	0.418	0.419	0.419	0.419
	FST_SES-ISA	0.595	0.392	0.408	0.390	0.387	0.359	0.389
	FST_SES-ADI	0.311	0.005 (*)	0.005 (*)	0.006 (*)	0.041	0.025	0.008 (*)
	FST_DRA-ISA	0.109	0.001 (**)	0.001 (*)	0.006 (*)	0.984	0.457	0.001 (*)
	FST_DRA-ADI	0.469	0.172	0.132	0.132	0.793	0.735	0.282
	FST_ISA-ADI	0.446	0.184	0.154	0.097	0.768	0.703	0.332
	LIK_SES-DRA	4.068	0.057	0.053	0.053	0.085	0.080	0.070
	LIK_SES-ISA	4.078	0.065	0.123	0.060	0.095	0.087	0.079
	LIK_SES-ADI	2.816	0.002 (*)	0.004 (*)	0.010 (*)	0.008 (*)	0.007 (*)	0.003 (*)
	LIK_DRA-SES	3.689	0.109	0.102	0.101	0.171	0.157	0.133
	LIK_DRA-ISA	0.809	0.001 (*)	0.003 (*)	0.014 (*)	0.833	0.498	0.020
	LIK_DRA-ADI	2.364	0.008 (**)	0.000 (***)	0.000 (***)	0.884	0.750	0.099
	LIK_ISA-SES	3.768	0.159	0.219	0.147	0.241	0.228	0.190
	LIK_ISA-DRA	0.833	0.001 (*)	0.001 (*)	0.014 (*)	0.871	0.526	0.018
	LIK_ISA-ADI	2.151	0.056	0.021	0.000 (***)	0.854	0.749	0.212
	LIK_ADI-SES	2.755	0.004 (*)	0.009 (*)	0.018	0.005 (*)	0.005 (*)	0.005 (*)
	LIK_ADI-DRA	3.743	0.156	0.010 (*)	0.010 (*)	0.802	0.776	0.437
	LIK_ADI-ISA	3.401	0.208	0.152	0.001 (*)	0.718	0.697	0.462
	V2P_SES-DRA	25.253	0.035	0.035	0.034	0.034	0.035	0.035
	V2P_SES-ISA	24.029	0.028	0.122	0.028	0.028	0.029	0.028
	V2P_SES-ADI	24.245	0.124	0.310	0.331	0.039	0.047	0.102
	V2P_DRA-ISA	4.093	0.167	0.033	0.254	0.779	0.768	0.477
	V2P_DRA-ADI	25.104	0.266	0.024	0.023	0.232	0.247	0.313
	V2P_ISA-ADI	23.700	0.298	0.279	0.019	0.213	0.229	0.311
	DAS_SES-DRA	0.081	0.904	0.904	0.903	0.904	0.903	0.903
	DAS_SES-ISA	0.069	0.867	0.785	0.866	0.866	0.864	0.866
	DAS_SES-ADI	0.304	0.998 (*)	0.995 (*)	0.990 (*)	0.998 (*)	0.998 (*)	0.997 (*)
	DAS_DRA-ISA	0.608	0.996 (*)	0.995 (*)	0.975	0.433	0.526	0.942
	DAS_DRA-ADI	0.177	0.909	0.997 (*)	0.997 (*)	0.256	0.285	0.679
	DAS_ISA-ADI	0.187	0.788	0.852	0.998 (*)	0.272	0.282	0.537

Table S1 (continued)

(b)	Summary statistics	observed value	A	B	C	A_{recent}	$A_{historical}$	$A_{ancient}$
Posterior probability [C.I.]			0.874 [0.804; 0.945]	0.007 [0.002; 0.012]	0.119 [0.051; 0.186]	0.000 [0.000; 0.000]	0.995 [0.991; 0.999]	0.005 [0.001; 0.009]
Summary statistics not used for model selection but used in model checking	HET_SES	0.404	0.195	0.400	0.195	0.280	0.210	0.370
	HET_DRA	0.336	0.270	0.370	0.330	0.435	0.360	0.340
	HET_ISA	0.361	0.220	0.220	0.350	0.560	0.315	0.310
	HET_ADI	0.539	0.400	0.620	0.420	0.680	0.310	0.430
	VAR_SES	17.281	0.940	0.960	0.930	0.940	0.960	0.960
	VAR_DRA	3.610	0.550	0.570	0.535	0.710	0.540	0.580
	VAR_ISA	4.080	0.550	0.050	0.580	0.770	0.590	0.595
	VAR_ADI	22.199	0.170	0.980 (*)	0.930	0.130	0.080	0.100
	DM2_SES-DRA	75.614	0.000 (***)	0.000 (***)	0.000 (***)	0.010 (*)	0.010 (*)	0.010 (*)
	DM2_SES-ISA	69.698	0.000 (***)	0.000 (***)	0.000 (***)	0.000 (***)	0.010 (*)	0.000 (***)
	DM2_SES-ADI	16.574	0.120	0.240	0.130	0.190	0.080	0.140
	DM2_DRA-ISA	0.985	0.460	0.120	0.730	0.860	0.740	0.360
	DM2_DRA-ADI	47.795	0.120	0.000 (***)	0.000 (***)	0.010 (*)	0.010 (*)	0.040
	DM2_ISA-ADI	41.057	0.090	0.000 (***)	0.000 (***)	0.010 (*)	0.010 (*)	0.030
	N2P_SES-DRA	6.500	0.320	0.465	0.320	0.395	0.400	0.515
	N2P_SES-ISA	6.500	0.210	0.375	0.260	0.605	0.270	0.445
	N2P_SES-ADI	7.417	0.350	0.690	0.440	0.515	0.310	0.490
	N2P_DRA-ISA	5.000	0.435	0.440	0.615	0.935	0.620	0.590
	N2P_DRA-ADI	6.833	0.505	0.265	0.175	0.740	0.485	0.565
	N2P_ISA-ADI	6.917	0.455	0.340	0.130	0.860	0.420	0.550
	H2P_SES-DRA	0.606	0.125	0.235	0.205	0.250	0.220	0.195
	H2P_SES-ISA	0.624	0.125	0.135	0.230	0.435	0.195	0.225
	H2P_SES-ADI	0.595	0.120	0.145	0.080	0.220	0.050	0.155
	H2P_DRA-ISA	0.370	0.210	0.290	0.325	0.520	0.360	0.220
	H2P_DRA-ADI	0.632	0.315	0.065	0.050	0.290	0.205	0.265
	H2P_ISA-ADI	0.634	0.270	0.080	0.040	0.360	0.165	0.240

Table S2 Prior distribution of temporal, demographic and mutation model parameters used in the two steps of ABC analysis. t_n (time of split, merge or admixture) is expressed in generations; N_X (effective population size) is expressed in number of diploid individuals and it was considered independent for each population; the prior distribution of N_X was chosen according to estimated effective population sizes obtained in Swatdipong *et al.* (2010) and according to our estimates of N_e obtained with Colony (Wang 2012) (data not shown) and after testing for demographic stability with Bottleneck (Cornuet & Luikart 1997) and M_P_val (Garza & Williamson 2001) (no evidence of bottlecks detected with both softwares, data not shown); r_{XtoY} (rate of admixture) represents the contribution of a population X to the origin of a new admixed population Y, ranging from 0 to 1; μ_{SSR} (mean mutation rate) is expressed in number of mutation per nucleotide per generation; P_{SSR} is the mean parameter of the geometric distribution of the length in number of repeats of mutation events. According to prior distribution of parameters, we imposed conditions on them (t_n) in order to achieve correct scenarios. All the probability distributions of the above parameters were set to Uniform.

	**First step**		
**Parameter**	**A**	**B**	**C**
t_1	1 – 25,000	1 – 25,000	1 – 25,000
t_2	1 – 25,000	-	-
t_3	2,500 – 250,000	2,500 – 250,000	2,500 – 250 000
t_4	250 001 – 1,250,000	250 001 – 1,250,000	250 001 – 1,250,000
N_X	1 – 1,000	1 – 1,000	1 – 1,000
r_{XtoY}	0.000 – 1.000	0.000 – 1.000	-
μ_{SSR}	$5 \cdot 10^{-5} - 1 \cdot 10^{-3}$	$5 \cdot 10^{-5} - 1x10^{-3}$	$5 \cdot 10^{-5} - 1 \cdot 10^{-3}$
P_{SSR}	0.000 – 0.600	0.000 – 0.600	0.000 – 0.600
conditions:	$t_1 \leq t_2$ and $t_1 \leq t_3$	$t_1 \leq t_3$	-

	**Second step**		
**Parameter**	**A_{recent}**	**$A_{historical}$**	**$A_{ancient}$**
$t_{1recent}$	1 – 10	-	-
$t_{2recent}$	1 – 10	-	-
$t_{1historical}$	-	11 – 200	-
$t_{2historical}$	-	11 – 200	-
$t_{1ancient}$	-	-	201 – 25,000
$t_{2ancient}$	-	-	201 – 25,000
t_3	2,500 – 250,000	2,500 – 250,000	2,500 – 250,000
t_4	250,001 – 1,250,000	250,001 – 1,250,000	250,001 – 1,250,000
N_X	1 – 1,000	1 – 1,000	1 – 1,000
$r_{UNStoADI}$	0.000 – 1.000	0.000 – 1.000	0.000 – 1.000
μ_{SSR}	$5 \cdot 10^{-5} - 1 \cdot 10^{-3}$	$5 \cdot 10^{-5} - 1 \cdot 10^{-3}$	$5 \cdot 10^{-5} - 1 \cdot 10^{-3}$
P_{SSR}	0.000 – 0.600	0.000 – 0.600	0.000 – 0.600
conditions:	$t_{1recent} \leq t_{2recent}$ and $t_{1recent} \leq t_3$	$t_{1historical} \leq t_{2historical}$ and $t_{1\,historical} \leq t_3$	$t_{1ancient} \leq t_{2ancient}$ and $t_{1ancient} \leq t_3$

Table S3 Parameter posterior distributions inferred by ABC under the A and $A_{historical}$ scenarios, respectively selected in first and second step. Mean, median and mode of the posterior distributions of each demographic, temporal and mutation model parameter are reported, together with their 90% and 95% Credibility Intervals. Posterior distribution of parameters was calculated using ln-tg transformation and logistic regression on the closest 0.1% simulated datasets. Time (t_n) is expressed in number of generations and effective population size (N_X) is expressed in number of diploid individuals. Populations codes: SES = Sesia; UNS = unsampled population; ADI = Adige; ISA = Isarco; DRA = Drava. $r_{UNStoADI}$: rate of admixture from population UNS the new admixed population ADI.

**First step**	**scenarioA**						
**Parameter**	**mean**	**median**	**mode**	**q025**	**q050**	**q950**	**q975**
N_{SES}	791	821	845	425	507	972	984
N_{UNS}	464	443	197	31	51	936	967
N_{ADI}	642	658	678	215	278	954	977
N_{ISA}	709	730	713	309	379	962	978
N_{DRA}	531	521	487	158	200	905	948
t_1	146	108	58	21	29	348	453
t_2	363	187	93	53	64	911	1,520
t_3	17,600	7,590	3,570	2,660	2,870	70,100	117,000
t_4	637,000	580,000	373,000	265,000	281,000	1,160,000	1,200,000
$r_{UNStoADI}$	0.681	0.717	0.767	0.217	0.314	0.906	0.933
μ_{SSR}	$6.14 \cdot 10^{-4}$	$6.31 \cdot 10^{-4}$	$6.51 \cdot 10^{-4}$	$2.22 \cdot 10^{-4}$	$3.02 \cdot 10^{-4}$	$8.76 \cdot 10^{-4}$	$9.11 \cdot 10^{-4}$
P_{SSR}	0.654	0.696	0.743	0.245	0.372	0.787	0.794

**Second step**	**scenario $A_{historical}$**						
**Parameter**	**mean**	**median**	**mode**	**q025**	**q050**	**q950**	**q975**
N_{SES}	701	719	732	326	397	951	975
N_{UNS}	629	659	835	125	195	959	979
N_{ADI}	461	427	332	82	115	913	953
N_{ISA}	752	777	795	372	447	965	982
N_{DRA}	460	425	337	143	172	867	932
$t_{1historical}$	43	34	16	3	5	112	132
$t_{2historical}$	117	116	102	34	43	191	196
t_3	11,400	5,590	3,180	2,550	2,710	35,200	63,700
t_4	706,000	680,000	288,000	269,000	288,000	1,190,000	1,220,000
$r_{UNStoADI}$	0.814	0.826	0.837	0.637	0.692	0.895	0.914
μ_{SSR}	$5.93 \cdot 10^{-4}$	$5.95 \cdot 10^{-4}$	$5.31 \cdot 10^{-4}$	$2.65 \cdot 10^{-4}$	$3.13 \cdot 10^{-4}$	$8.72 \cdot 10^{-4}$	$9.17 \cdot 10^{-4}$
P_{SSR}	0.720	0.748	0.770	0.460	0.559	0.794	0.797

Fig. S1 Midpoint-rooted Maximum-Likelihood Phylogeny of 83 *T. thymallus* mtDNA CR haplotypes based on the ML algorithm implemented in PhyML v3.0 (Guindon *et al.* 2010) by using best-fit substitution model HKY + I + G (Hasegawa *et al.* 1985) (transition:transversion = 3.2906; proportion of invariable sites (I) = 0.8130; gamma distribution shape parameter (G) = 0.9010) selected by AICc and BIC with jModeltest 2.1.1. (Darriba *et al.* 2012). Haplotypes marked with * are the ones retrieved in the present dataset. GenBank sequences of *T. grubei*, *T. brevirostris* and *T. arcticus* served as outgroup taxa. Bootstrap percentages ≥ 70 are reported at nodes. Nomenclature of major mtDNA lineages (according to Weiss *et al.* 2002) is included.

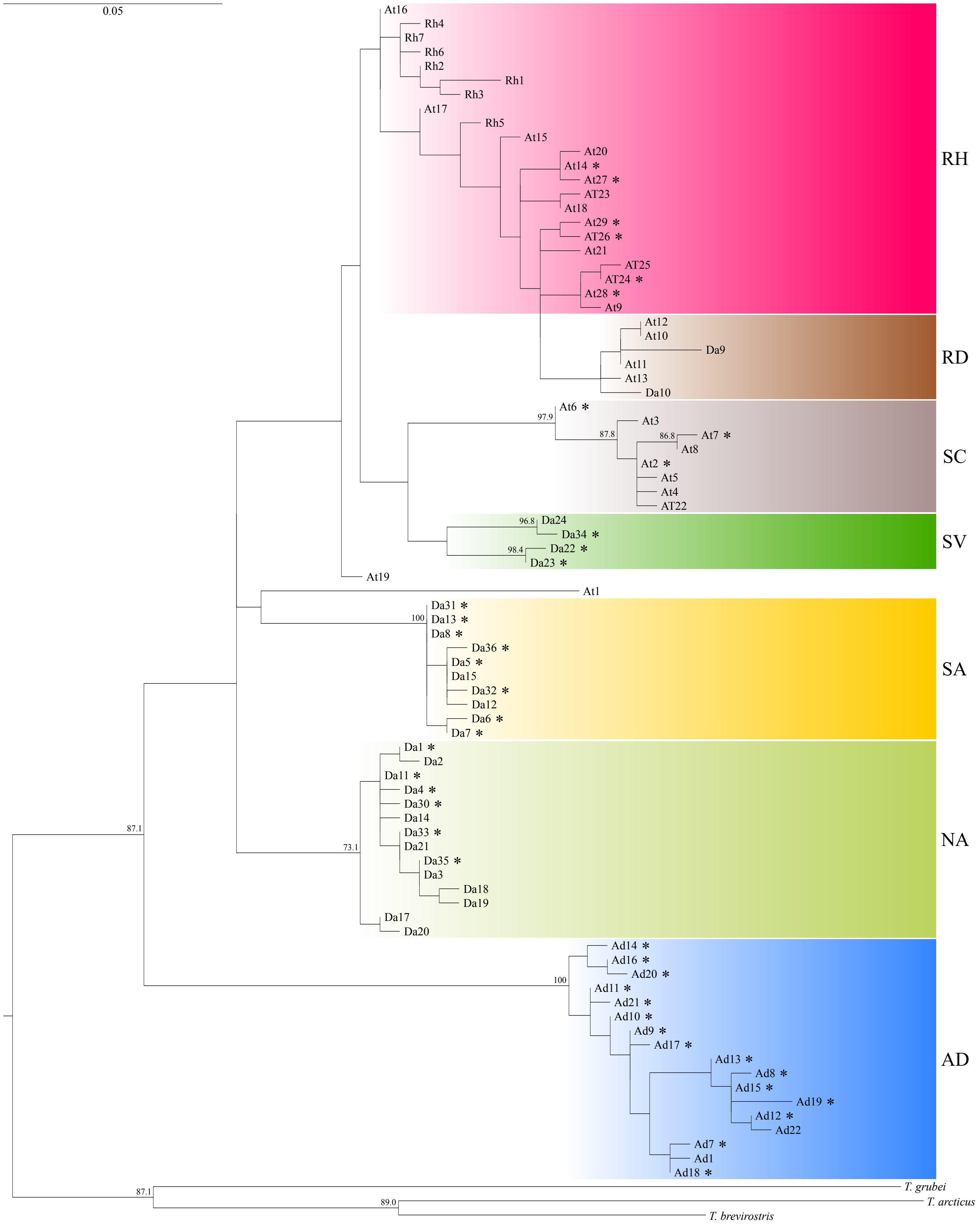

Fig. S2 Intra-cluster genetic substructure analysis results. For each cluster identified in the first-level STRUCTURE analysis (see Fig. 2), only individuals with a q-value ≥ 0.90 for that specific cluster were included in the analysis (qAD1, qAD2, qNA, qRH1, qSA, qSC, qSV; qRH2 was not analysed because it was under-represented in the test-samples and completely absent in the reference-samples). For each cluster, mean of the estimated ln probability of data (± st. dev.) (black dots and lines) and the ΔK statistics (Evanno *et al.* 2005; red line) for the different tested number of STRUCTURE genetic clusters (K) are shown on top, for both correlated- and independent allele frequencies models. Population structure for most likely K values, ordered by sampling sites (codes according to IDs in Table 1) are shown below, for both correlated- and independent allele frequencies models. Each vertical bar represents a fish and its proportional membership to one of the genetic clusters inferred by STRUCTURE.

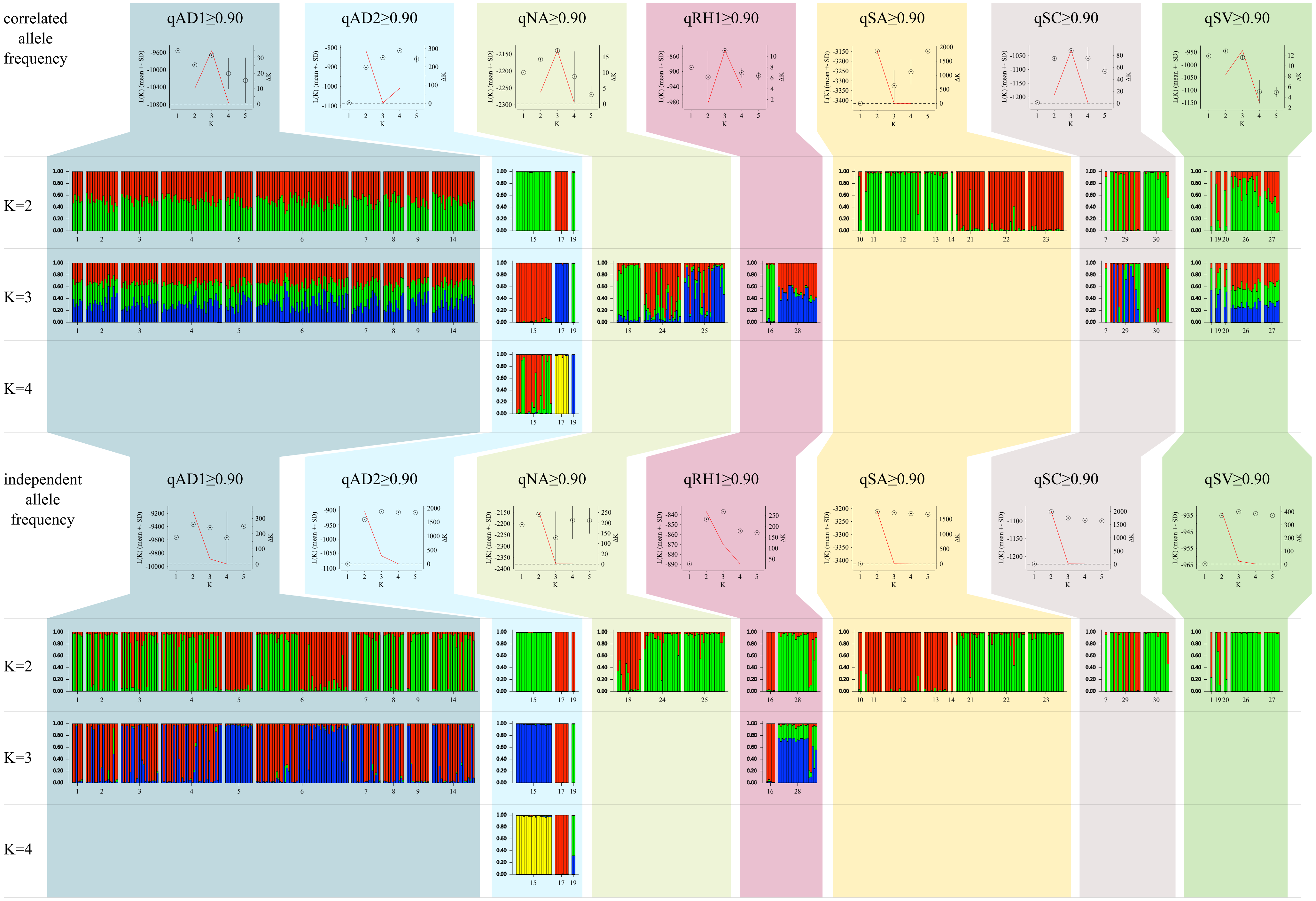

Supplementary References

Cornuet JM, Luikart G (1997) Description and power analysis of two tests for detecting recent population bottlenecks from allele frequency data. *Genetics*, **144**, 2001-2014.

Darriba D, Taboada G, Doallo R, Posada D (2012) jModelTest 2: more models, new heuristics and parallel computing. *Nature Methods*, **9**, 772.

Evanno G, Regnaut S, Goudet J (2005) Detecting the number of clusters of individuals using the software STRUCTURE: a simulation study. *Molecular Ecology*, **14**, 2611-2620.

Garza JC, Williamson EG (2001) Detection of reduction in population size using data from microsatellite loci. *Molecular Ecology*, **10**, 305-318.

Gelman A, Carlin JB, Stern HS, Rubin DB (1995) *Bayesian Data Analysis*. Chapman & Hall, London.

Goldstein DB, Linares AR, Cavalli-Sforza LL, Feldman MW (1995) Genetic absolute dating based on microsatellites and the origin of modern humans. *Proceedings of the National Academy of Sciences of the United States of America*, **92**, 6723-6727.

Guindon S, Dufayard JF, Lefort V, Anisimova M, Hordijk W, Gascuel O (2010) New algorithms and methods to estimate maximum-likelihood phylogenies: assessing the performance of PhyML 3.0. *Systematic Biology*, **59**, 307-321.

Hasegawa M, Kishino H, Yano T (1985) Dating of the human-ape splitting by a molecular clock of mitochondrial DNA. *Journal of Molecular Evolution*, **22**, 160-174.

Jin L, Chakraborty R (1994) Estimation of genetic distance and coefficient of gene diversity from single-probe multilocus DNA fingerprinting data. *Molecular Biology and Evolution*, **11**, 120-127.

Nei M (1987) *Molecular Evolutionary Genetics*. Columbia University Press, New York.

Pascual M, Chapuis MP, Mestres F, Balanya J, Huey RB, Gilchrist GW *et al.* (2007) Introduction history of *Drosophila subobscura* in the New World: a microsatellite-based survey using ABC methods. *Molecular Ecology*, **16**, 3069-3083.

Wang J (2012) Computationally efficient sibship and parentage assignment from multilocus marker data. *Genetics*, **191**, 183-194.

Weir BS, Cockerham C (1984) Estimating F-statistics for the analysis of population structure. *Evolution*, **38**, 1358-1370.
